# Reversible splenial lesion syndrome with mental disorders as only manifestation

**DOI:** 10.1186/s12883-021-02391-6

**Published:** 2021-09-15

**Authors:** Ziyun Xu, Zhaoguang Zhou, Wentao Jang, Jianhong Tian, Gangqiang Hou

**Affiliations:** Department of Radiology, Shenzhen Mental Health Center, Shenzhen Kangning Hospital, 518020 Shenzhen, China

**Keywords:** Reversible splenial lesion syndrome, Mental disorders, Magnetic resonance imaging

## Abstract

**Background:**

Reversible splenial lesion syndrome (RESLES) was reported to be associated with variable entities. However, much less is known about the cases in which the mental disorders act as the only manifestation.

**Method:**

Total ten patients of RESLES were obtained in this retrospective study from Shenzhen Kangning Hospital. T1-fluid attenuated inversion recovery (T1-FLAIR), T2-weighted images, T2-FLAIR, diffusion-weighted images and apparent diffusion coefficient map were performed on all the patients. Clinical manifestations, laboratory examination results, magnetic resonance imaging (MRI) findings, treatments and outcomes were analyzed.

**Result:**

All patients showed different mental disorders as the only manifestation. There were two cases of alcohol abuse, one of Asperger’s syndrome with malnutrition, one of infection and one of invasive pituitary adenoma. The other cases were diagnosis as major depressive disorder, dissociative and conversion disorders, undifferentiated somatoform disorder, unspecified psychosis and bipolar disorder, respectively. Three patients were completely recovered while the clinical symptoms of rest seven patients partially recovered at the follow-up three months later. Oval-shaped lesion centered on the splenial of corpus callosum (SCC) was observed in all patients using MRI. The lesions of SCC of all patients were completely resolved within five weeks.

**Conclusions:**

We found that RESLES might only showed mental symptoms. On the one hand, for the patients with acute mental disorders, clinicians should be alert to the possibility of RESLES caused by physical disease. On the other hand, we suggest that mental disorder might be a precipitating factor of RESLES.

## Introduction

Reversible splenial lesion syndrome (RESLES) defined as clinico-radiological syndrome is identified by transient localized lesions in the splenium of the corpus callosum (SCC) on magnetic resonance imaging (MRI). It can be seen in a wide variety of clinical conditions including seizures [1, 2], drug therapy [3] or withdrawal [4], viral encephalitis [5], hypoglycemic encephalopathy [6], eclampsia [7], Marchiafava-Bignami disease (MBD) [8], hypernatremia and hypoglycemia [9], high-altitude cerebral edema [9], meningitis [10], bacterial [11] and neoplasm [12]. However, the pathophysiological mechanism still remains to be understood.

It is well known that corpus callosum is closely related to behavior. Literatures have reported that the occurrence of neuropsychological disorders was probably secondary to lesions of corpus callosum [13]. RESLES exhibits diverse clinical manifestations and sometimes shows neurological symptoms [14]. The most common neurological symptoms include delirious behavior and consciousness disturbance. Indeed, the neurological symptoms are always accompanied by prodromal symptoms such as fever, headache and seizures [5, 15]. However, the cases in our research were admitted to hospital only for altered mental status. As far as we know, little attention has been given to such cases in previous literatures.

To better understand the exact correlation between RESLES and mental disorders, we retrospectively analyze 10 cases of RESLES with mental disorder as the only manifestation including their clinical manifestation, MRI features, laboratory characteristics, treatment and outcome.

## Materials and methods

### Subjects

Cases were firstly selected by searching through Picture Archiving and Communication Systems using the keywords “splenium of corpus callosum” and the time range from December, 2017 to January, 2021. Then, two radiologists who had at least 5 years of experience with neuro-radiologic imaging read the images again to confirm the diagnosis of RESLES. Finally, ten RESLES met the criteria and were included in our study. The study was approved by the ethics committee of Shenzhen Kangning Hospital (2020-K010-01), and informed consent was written by the patients or their legal guardians.

### Criteria for RESLES

According to Garcia-Monco [16], the patients were obtained through the following criteria: (1) showing neuropsychiatric symptoms; (2) showing lesions in typical locations on MRI (Type I, an isolated round/oval lesion centered on splenium of SCC; Type II, an extended lesion to the adjacent white matter based on Type I. Type III, a lesion at center of posterior corpus callosum extending to the anterior corpus callosum [14].); (3) both the lesions and clinical symptoms were reversible.

### Image Collection

MRI was performed on a 3T magnetic resonance system with the parameters of transverse T1-fluid attenuated inversion recovery (T1-FLAIR), transverse T2-weighted images (T2WI), transverse T2-FLAIR, DWI (echo-planar imaging sequence; b-value = 0 and 1000 s/mm; TR = 4600 ms; TE = 65.5 ms; slice thickness = 5.0 mm; intersection gap = 1.5 mm; slice number = 20; FOV = 240 × 240 mm; matrix = 160 × 160) and ADC map.

### Data analysis

Data of patients were collected via medical records and the patients were followed up by telephone three months after being discharged. Clinical data including the clinical manifestations, laboratory examination results, MRI findings, drug use, and outcomes were reviewed by all authors.

## Result

### Clinical features

The patients were admitted to our hospital for the altered mental status and were diagnosed as different mental disorders according to ICD-10 (Table 1). The age ranged from 14 to 51 with a mean value of 28.7 (± 9.6). All the subjects did not have a fever, vomiting, seizures, trauma,hypertension or loss of consciousness. Case 3 had a seriously irregular intake and developed malnutrition. Case 4 had a history of maculopapular. Case 5 had invasive pituitary adenoma. Case 7 delivered a week before the onset.

**Table 1:** Clinical data of the patients with RESLES

Patient ID	Age/ gender	Clinical symptoms	Clinical diagnosis	Treatment	Outcome (hospital stays, days)	
1	34/M	alcohol dependence/refusal of food intake/depressive	Alcohol dependence with withdrawal	Diazepam/ Oxazepam/ Mirtazapine/ Vitamin B complex		CR (13)
2	26/M	alcohol dependence/depressive/auditory hallucination/delusion	Alcohol dependence with alcohol-induced psychotic disorder	Diazepam/ Paliperidone/ Valproate/ Vitamin B complex		CR (9)
3	28/M	sensitive/suspicious/loneliness/impaired personal hygiene/irregular intake	Asperger’s syndrome	Aripiprazole/ Mirtazapine/ Vitamin B complex		PR (27d)
4	14/M	behavioral abnormalities/irrelevant talking/auditory hallucination	Other specified mental disorders due to known physiological condition	Olanzapine/ Clonazepam		T/CR (5d)
5	51/F	delusion/ auditory hallucination/ depressive	Other specified mental disorders due to known physiological condition	Quetiapine/ Lorazepam		T/PR (2d)
6	20/F	stupor/depressive	Major depressive disorder, single episode, severe with psychotic features	Quetiapine/ Escitalopram		T/PR (2d)
7	28/F	depressive/behavioral abnormalities/ irrelevant talking/disturbed sleep	Dissociative and conversion disorders	Olanzapine		PR (26d)
8	27/M	persistent headache/	undifferentiated somatoform disorder	Duloxetine / Valproate / Olanzapine		PR (43d)
9	28/M	delusion/behavioral abnormalities/disturbed sleep	Unspecified psychosis not due to a substance or known physiological condition	Ziprasidone / Quetiapine		PR (26)
10	31/F	elevated mood/decreased need for sleep/greater talkativeness	Bipolar disorder, current episode manic severe with psychotic features	Quetiapine/ Lithium carbonate		PR (14d)

*MBD* Marchiafava-Bignami disease; *CR* completely recovered. *T* Transferred to other hospital; *PR* partial recovered; *F *female; *M* male

### Laboratory examinations

One case initially showed decreased serum vitamin B12 level (case 3; 112 pmol/L), while two cases showed increased serum vitamin B12 level (case 1 and case 2; 926 pmol/L; >1107 pmol/L) after treatment of Vitamin B complex (Table 2). There were no findings of virus or bacteria.

**Table 2:** Examinations of the patients with RESLES

Patient ID	Biochemical blood tests								CSF
	WBC (10^9/L)	LY (10^9/L)	NEUT (10^9/L)	CPR(mg/l)	Serum vitamin B12(pmol/L)	Glu (mmol/L)	Na^+^(mmol/L)	K^+^(mmol/L)	
1	9.88	3.1	6.2	NE	926↑	3.72	138.6	4.58	NE
2	6.02	2	3.7	NE	> 1107↑	6.1	140.3	3.54	NE
3	9.89	1.4	5.4	0.16	112↓	6.05	142.2	3.65	NE
4	9.21	2.3	5.8	0.15	NE	NE	140.6	3.57	NE
5	6.02	1.4	4	NE	NE	NE	NE	NE	NE
6	7.96	1.1	5.9	1.84	NE	4.21	141.3	3.7	NE
7	9.52	1.8	6.2	3	NE	4.8	141.5	3.53	NE
8	5.22	1.6	2.8	2.5	NE	5.19	143.2	3.56	NE
9	5.59	1.6	3.4	9.23	NE	5.25	143.7	3.57	Normal
10	5.31	1.2	3.6	0.704	NE	4.68	140.2	4.02	NE

*CSF* cerebrospinal fluid, *NE* not examined

### MRI findings

**Fig. 1:**
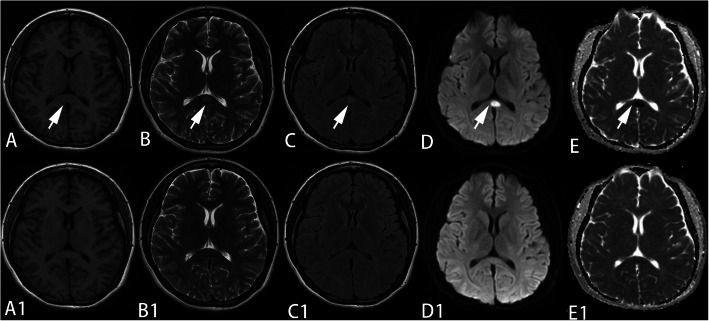
The MRI findings of case 7. It showed oval-shaped center on SCC characterized by hypo-intensity on T1-FLAIR (**A**), hyper-intensity on T2WI (**B**), T2-FLAIR (**C**) and DWI (**D**) and low ADC value (**E**). The lesions were resolved in the follow-up (**A1**-**E1**)

**Fig. 2:**
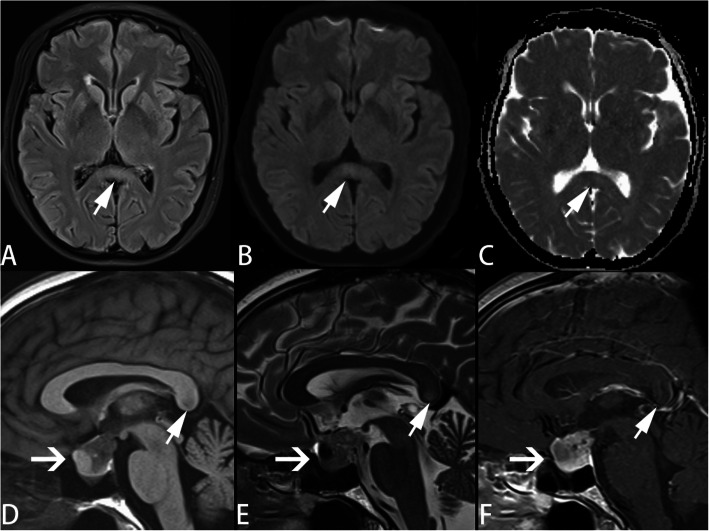
The MRI finding of case 5. The arrow (➚) showed lesion center on SCC characterized by hypo-intensity on T1-FLAIR (**D**), hyper-intensity on T2WI (**E**), T2-FLAIR (**A**) and DWI (**B**) and low ADC value (**C**). Signal of lesion center on SCC showed no enhancement (**F**). The lesions of SCC were resolved at telephone follow-up 15 days later. Moreover, there was a large oval-shaped lesion in the sellar region with a size of about 18 × 19 × 24mm (**D** & **E**; ➜). The abnormal signal was mixed. The lesion showed uneven enhancement (**F**). Pathological diagnosis confirmed pituitary adenoma

All the patients were checked using MRI within one week after hospitalization. The lesions of all patients were type I, showing the oval-shaped lesions in SCC on MRI (Table 3). The abnormal signals were characterized by hypo-intensity on T1-FLAIR, hyper-intensity on T2WI, T2-FLAIR and DWI and low ADC value (Fig. 1). All the lesions of SCC had resolved when the patients were re-scanned within 5 weeks (Table 3). Moreover, case 5 showed a large oval-shaped lesion in the sellar region (Fig. 2). Pathological diagnosis confirmed the diagnosis of pituitary adenoma of case 5.

### Treatment and outcome

The patients received treatments of psychotropic drugs during the hospital day (Table 1). Three patients (case 4, 5 and 6) showed no improvement after a short period of treatment and then they were transferred to hospital for infection, pituitary adenoma and high risk of pulmonary embolism, respectively. The other patients showed notably improvement of clinical symptoms after treatment. Three cases (case 1, 2 and 4) completely recovered, while the mental status of rest seven cases partially recovered in the follow-up. The lesions on MRI were completely resolved within five weeks.

**Table 3:** MRI findings of patients with RESLES

Patient ID	Initial MRI							Follow-up	
	Interval after the onset of mental symtoms(days)	Shape of splenial lesion	T1-FLAIR	T2WI	T2-FLAIR	DWI	ADC value (10^-3^mm^2^/s)	Reversal	Interval after the initial MRI (days)
1	6	oval	sl L	sl H	sl H	H	0.376	Y	35
2	8	oval	I	sl H	sl H	H	0.68	Y	15
3	10	oval	L	sl H	sl H	H	0.672	Y	17
4	5	oval	L	H	H	H	0.378	Y	18
5	~ 60	oval	sl L	sl H	sl H	sl H	0.698	Y	15
6	~ 120	oval	L	sl H	sl H	H	0.558	Y	23
7	13	oval	sl L	sl H	H	H	0.262	Y	15
8	~ 38	oval	sl L	sl H	sl H	H	0.365	Y	10
9	~21	oval	sl L	sl H	sl H	H	0.389	Y	22
10	~60	oval	sl L	sl H	sl H	H	0.389	Y	20

*I* isointense; *L* low signal; *sl L* slightly low signal; *H* high signal; *sl H* slightly high signal; ~, approximately equal to;

## Discussion

The corpus callosum is composed of densely bundled white matter tracts and plays an important role in interhemispheric communication and coordination. It is divided into four parts: the rostrum, the genu, the body, and the splenium. Characterized by a higher density of receptors including cytokine receptors, glutamate and other excitatory amino acid receptors, toxin receptors, and drug receptors [17–19], the corpus callosum, particularly the splenium, is more vulnerable to cytotoxic edema than other brain areas [1, 20, 21].

The terms of “mild encephalopathy with reversible splenial lesions (MERS),” “reversible/transient splenial lesions,” “clinically silent lesions in the splenium of the corpus callosum,” “transient focal lesions in the splenium of the corpus callosum” and “cytotoxic lesions of the corpus callosum” which are similar to RESLES were reported in a wide spectrum of clinical conditions. These conditions were classified as malignancy, infections, trauma, metabolic abnormalities,drug therapy, subarachnoid hemorrhage (SAH) and other entities [14].

Consistent to the previous findings, three cases in our study developed metabolic abnormalities. MBD is one of the forms of metabolic abnormalities [8]. The pathogenesis of MBD is associated with chronic alcoholism, nutritional deficiency and metabolic disorders [8, 22]. Consistently, both of our case 1 and case 2 are diagnosed as alcohol dependence. Long-term alcohol might lead to severe malnutrition (especially lack of B vitamins). Case 3 was diagnosed as Asperger’s syndrome and showed decreased serum vitamin B12 level. He had a seriously irregular intake, and thus developed serious malnutrition, which may explain his markedly decreased serum vitamin B12 level. Given that there is no evidence of an association between Asperger’s syndrome and RESLES, we believe that malnutrition is more likely to be the cause. The alcohol abuse and malnutrition increase the susceptibility to injury pro-apoptotic drive and then lead to endothelial dysfunction, which result in the cytokines released from microglia and eventually cause the cytotoxic edema of corpus callosum [14, 23].

Although the cases of RESLES with other malignancies might be caused by chemotherapy [12, 24], the RESLES was also found in CNS malignancies before treatment [12, 14]. Case 5 had pituitary adenomas and did not get any treatment at the onset. The effect of the pituitary adenomas on RESLES has not been determined. In some cases of CNS malignancies, the author suggests that the RESLES is attributed to a release of cytokines into the cerebrospinal fluid resulted by infiltration of malignant cells [13]. Also, we speculate that the metabolic abnormality caused by endocrine disorders is likely to be a precipitating factor of RESLES with pituitary adenomas. However, without exact evidences, further researches are needed to reveal the underlying mechanism.

On the one hand, the patients with RESLES may only showed mental symptoms, which was never reported in previous studies. RESLES triggered by infection often occurs in children and young adults [5, 16]. The virus is the main pathogen [16], as well as the bacteria [11], mycoplasma pneumoniae [25] and plasmodium [26] are also reported as the causes of RESLES. Similar to previous reports, our case 4 had a history of infection in forms of recurrent of maculopapular. Differently, the case 4 only showed altered mental states without any prodromal manifestations, while some manifestations, such as fever, digestive tract symptoms and cough, were reported to preceded the appearance of neurological symptoms in previous reports [5]. Moreover, the antipsychotic treatment for him was not working but anti-inflammatory treatment was effective. It indicated that etiological therapy might be more effective for RESLES caused by infections. Similarly, the clinical symptoms of case 1–3 significantly improved after treatment of vitamin B complex. Therefore, it is important to be alert of RESLES thought the patients only showed mental symptoms to choose the effectively treatment.

On the other hand, the other five cases (case 6–10) are special owing to that they might give a new sight to identify the etiologies of RESLES. Firstly, most of the etiologies described above were ruled out through clinical history, examinations, and clinical diagnosis. There was no evidence of history of malignancy or chemotherapy, the symptom of infection (fever, leukocytosis, nuchal rigidity and history of travel to endemic areas), SAH (thunderclap headache), metabolic pathology (Fluid-electrolyte imbalances, cirrhosis or hepatic dysfunction, liver transplantation, malnutrition, or AIDS, Wernicke encephalopathy or Marchiafava-Bignami disease and Wilson disease) and Trauma. Secondly, they start with mental disorders without prodromal symptoms, and were clinically diagnosed with different mental disorders. Thirdly, the possibility of drug cause was also ruled out based on the remarkable improvement of symptoms after treatment of psychotropic drugs, although the drugs used in these cases like Olanzapine [27] have been reported as an inducement of RESLES. Thus, we suspect that RESLES of these cases might be caused by mental disorders. Moreover, we found that a case report about postpartum psychosis without any other physical diseases was similar to case 7. She presented two weeks postpartum with new onset of behavioral alteration and irrelevant talking without fever, headache, or seizures. A possibility of postpartum psychosis was considered to be the associated condition after ruling out various etiologies for RESLES [28]. Given the evidences, the mental disorders are inclined to be considered as the possible etiology of the RESLES.

There are three limitations in our study. Firstly, the sample is limited, since the patients were all from the same hospital and the simple size was relatively small. Further studies with a larger number of patients from multiple centers are necessary to confirm our results. Secondly, since the patients were diagnosed as different mental disorders, more homogenous sample is needed in the future researches. Lastly, restricted to retrospective studies, the role of nutritional deficiency or unidentified viral infection cannot be ruled out with certainty because of the lack of more detailed examination results.

## Conclusions

MRI findings are essential to identify the RESLES from the nonspecific clinical manifestations. We found that patients with RESLES might showed mental disorders as the only manifestation. It indicated that acute mental disorders might be the only clinical symptom of RESLES caused by infection or metabolic abnormalities. Clinicians should be aware of the precipitating factors and choose the accurate interventions. More importantly, we found that mental disorder might in turn to be a precipitating factor of RESLES, which was hardly reported before.

## Data Availability

The data that support the findings of this study are available from the corresponding author upon reasonable request.

## References

[1] Prilipko O, Delavelle J, Lazeyras F, Seeck M (2005). Reversible cytotoxic edema in the splenium of the corpus callosum related to antiepileptic treatment: report of two cases and literature review. Epilepsia..

[2] Polster T, Hoppe M, Ebner A (2001). Transient lesion in the splenium of the corpus callosum: three further cases in epileptic patients and a pathophysiological hypothesis. Journal of neurology, neurosurgery, and psychiatry..

[3] Renard D, Bonafe A, Heroum C (2007). Transient lesion in the splenium of the corpus callosum after oral corticoid therapy. European journal of neurology..

[4] Gürtler S, Ebner A, Tuxhorn I, Ollech I, Pohlmann-Eden B, Woermann FG (2005). Transient lesion in the splenium of the corpus callosum and antiepileptic drug withdrawal. Neurology..

[5] Chen WX, Liu HS, Yang SD (2016). Reversible splenial lesion syndrome in children: Retrospective study and summary of case series. Brain & development..

[6] Malik AM (2013). The reversible corpus callosum splenium lesion associated with hypoglycemic encephalopathy. The Neurohospitalist..

[7] Yang Q, Chang CC, Liu M, Yu YQ (2019). Sequential occurrence of eclampsia-associated posterior reversible encephalopathy syndrome and reversible splenial lesion syndrome (a case report): proposal of a novel pathogenesis for reversible splenial lesion syndrome. BMC medical imaging..

[8] Hillbom M, Saloheimo P, Fujioka S, Wszolek ZK, Juvela S, Leone MA (2014). Diagnosis and management of Marchiafava-Bignami disease: a review of CT/MRI confirmed cases. Journal of neurology, neurosurgery, and psychiatry..

[9] Garcia-Monco JC, Martínez A, Brochado AP, Saralegui I, Cabrera A, Beldarrain MG (2010). Isolated and reversible lesions of the corpus callosum: a distinct entity. J Neuroimaging.

[10] Oquist M, Farooq MU, Gorelick PB (2014). Restricted diffusion of the splenium of the corpus callosum in viral meningitis. The Neurohospitalist..

[11] Avcu G, Kilinc MA, Eraslan C, Karapinar B, Vardar F (2017). Mild encephalitis/encephalopathy with reversible splenial lesion (MERS) associated with Streptococcus pneumoniae Bacteraemia. Journal of infection and public health..

[12] Maeda M, Tsukahara H, Terada H (2006). Reversible splenial lesion with restricted diffusion in a wide spectrum of diseases and conditions. Journal of neuroradiology = Journal de neuroradiologie..

[13] Devinsky O, Laff R (2003). Callosal lesions and behavior: history and modern concepts. Epilepsy & behavior: E&B..

[14] Starkey J, Kobayashi N, Numaguchi Y, Moritani T (2017). Cytotoxic Lesions of the Corpus Callosum That Show Restricted Diffusion: Mechanisms, Causes, and Manifestations. Radiographics: a review publication of the Radiological Society of North America, Inc..

[15] Hayashi M, Sahashi Y, Baba Y, Okura H, Shimohata T (2020). COVID-19-associated mild encephalitis/encephalopathy with a reversible splenial lesion. Journal of the neurological sciences..

[16] Garcia-Monco JC, Cortina IE, Ferreira E (2011). Reversible splenial lesion syndrome (RESLES): what’s in a name?. J Neuroimaging.

[17] Hassel B, Boldingh KA, Narvesen C, Iversen EG, Skrede KK (2003). Glutamate transport, glutamine synthetase and phosphate-activated glutaminase in rat CNS white matter. A quantitative study. Journal of neurochemistry..

[18] Domercq M, Matute C (1999). Expression of glutamate transporters in the adult bovine corpus callosum. Brain research Molecular brain research..

[19] Goursaud S, Kozlova EN, Maloteaux JM, Hermans E (2009). Cultured astrocytes derived from corpus callosum or cortical grey matter show distinct glutamate handling properties. Journal of neurochemistry..

[20] Moritani T, Smoker WR, Sato Y, Numaguchi Y, Westesson PL (2005). Diffusion-weighted imaging of acute excitotoxic brain injury. AJNR American journal of neuroradiology..

[21] Takayama H, Kobayashi M, Sugishita M, Mihara B (2000). Diffusion-weighted imaging demonstrates transient cytotoxic edema involving the corpus callosum in a patient with diffuse brain injury. Clinical neurology and neurosurgery..

[22] Lee SH, Kim SS, Kim SH, Lee SY (2011). Acute Marchiafava-Bignami disease with selective involvement of the precentral cortex and splenium: a serial magnetic resonance imaging study. The neurologist..

[23] Takefuji S, Murase T, Sugimura Y (2007). Role of microglia in the pathogenesis of osmotic-induced demyelination. Experimental neurology..

[24] Tha KK, Terae S, Sugiura M (2002). Diffusion-weighted magnetic resonance imaging in early stage of 5-fluorouracil-induced leukoencephalopathy. Acta neurologica Scandinavica..

[25] Yuan ZF, Shen J, Mao SS (2016). Clinically mild encephalitis/encephalopathy with a reversible splenial lesion associated with Mycoplasma pneumoniae infection. BMC infectious diseases..

[26] Hantson P, Hernalsteen D, Cosnard G (2010). Reversible splenial lesion syndrome in cerebral malaria. Journal of neuroradiology = Journal de neuroradiologie..

[27] Kaino K, Kumagai R, Furukawa S (2017). Reversible splenial lesion syndrome with a hyperosmolar hyperglycemic state and neuroleptic malignant syndrome caused by olanzapine. Journal of diabetes investigation..

[28] Udaya SC, Chauhan BN, Philip VJ (2015). Bright splenium of a psychotic mind. Annals of Indian Academy of Neurology..

